# Nonadiabatic Couplings Can Speed Up Quantum Tunneling Transition Path Times

**DOI:** 10.1021/acs.jpclett.2c03008

**Published:** 2022-11-07

**Authors:** Tom Rivlin, Eli Pollak

**Affiliations:** Chemical and Biological Physics Department, Weizmann Institute of Science, 76100Rehovot, Israel

## Abstract

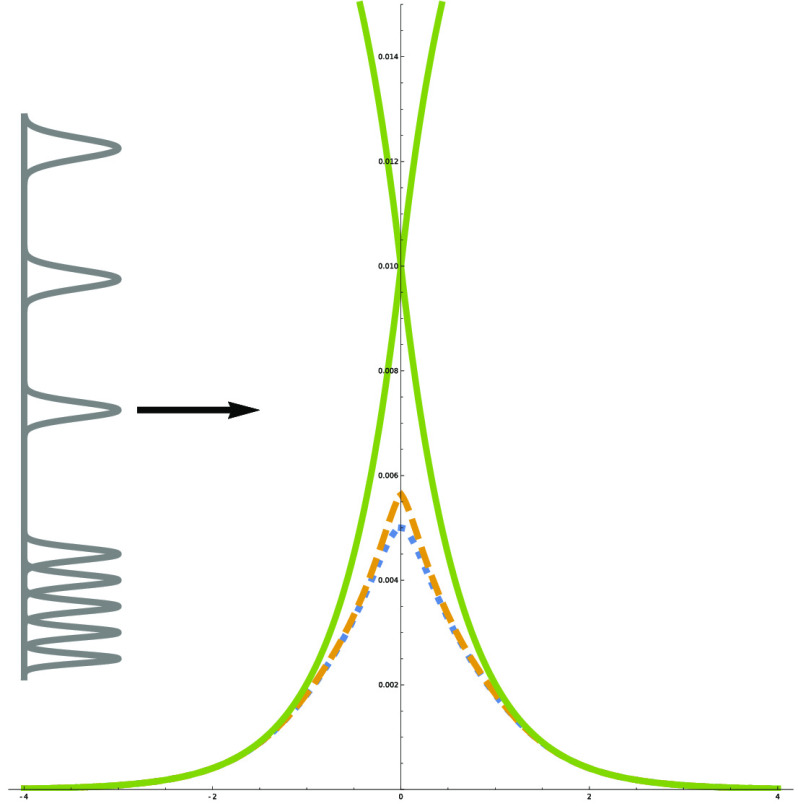

Quantum tunneling is known to play an important role in the dynamics of systems with nonadiabatic couplings. However, until recently, the time-domain properties of nonadiabatic scattering have been severely under-explored. Using numerically exact quantum methods, we study the impact that nonadiabatic couplings have on the time it takes to tunnel through a barrier. We find that the Wigner phase time is the appropriate measure to use when determining the tunneling flight time also when considering nonadiabatic systems. The central result of the present study is that in an avoided crossing system in one dimension, the nonadiabatic couplings speed up the tunneling event, relative to the adiabatic case in which all nonadiabatic coupling is ignored. This has implications for both the study of quantum tunneling times and for the field of nonadiabatic scattering and chemistry.

Nonadiabatic effects play important roles in many chemical reactions, and the dynamics of these systems has been studied extensively both quantum mechanically and otherwise.^1−17^ However, the time-domain properties of systems such as avoided crossings have received comparatively little attention.

In contrast, the temporal dynamics of atoms and molecules has been studied extensively. In the context of quantum tunneling much work has been aimed at understanding quantum tunneling times.^18−26^ These studies have uncovered various challenging nonclassical phenomena. One striking example is the MacColl–Hartman effect,^27,28^ whereby the tunneling time can become independent of the width of the barrier. The tunneling time question has raised an intense debate over the appropriate metrics to use to measure the time taken to tunnel. The tunneling time question is part of the more general question: What is the time scale associated with a quantum transition? This is relevant for example to such problems as electronic transition times^29−31^ and has been the subject of many experiments.^30,32−39^

Previously, we have analyzed tunneling times for one-dimensional, one-state systems,^40−48^ defining a metric called the tunneling flight time. This measure considers the time it takes particles characterized by incident Gaussian wavepackets to traverse a barrier and hit a screen. In the appropriate limit, we have previously shown^44^ that it is related to the Wigner phase time,^49,50^ which is based on time-independent scattering theory. One of the goals of the present study is to understand whether the same holds true when considering electronic transitions—whether the results we demonstrated in the one-state model also hold true when there are two potential energy curves.

Recently,^48^ we started exploring temporal properties of quantum transitions in nonadiabatic systems. We focused on a comparison between the numerically exact quantum determination of flight times and the results obtained with approximate quasi-classical methods such as surface hopping.^51−54^ These methods have a history of being applied to nonadiabatic systems^55,56^ as well as systems with non-Born–Oppenheimer corrections^57,58^ and tunneling.^59^ But in our previous work, we found them lacking in their ability to detect quantum effects such as tunneling and resonances in the time domain. This is despite there being an abundance of quantum effects in avoided crossing systems.^12,14−17^

The central challenge we address in this Letter is to understand how nonadiabatic interactions affect tunneling times. We consider a simple example of a two-state, one-dimensional, avoided crossing system.^60−62^ We investigate the change in tunneling time when nonadiabatic couplings are turned on and off: how does the interaction with the other potential energy surface affect the tunneling dynamics of the system?

The central result of this study is that nonadiabatic couplings can speed up quantum tunneling. We compare the tunneling flight time through the adiabatic barrier resulting from the avoided crossing to the tunneling flight time as determined numerically exactly when the nonadiabatic coupling is included. In demonstrating this, we also verify that the phase time and the tunneling flight time coincide in the appropriate limit for numerically exact diabatic systems with more than one potential energy curve.

The specific model system we study is the single avoided crossing (SAC) system of Tully^51^ (which he called the “simple” avoided crossing). We focus solely on quantum mechanical methods, approaching the problem using both time-dependent and time-independent scattering theory. We study the problem from the perspective of a Gaussian time-dependent wave packet with a finite momentum width incident on the avoided crossing, and also from the perspective of a time-independent, single-energy plane wave scattering solution. Previously we have established that for scattering on a single surface, in the limit of zero energy width of the incident wave packet, the tunneling flight time (calculated using time-dependent mechanics) coincides with the phase time (calculated using time-independent methods).^44,45^ Here we show that this result holds when including the nonadiabatic terms coupling the two states.

The time-independent Schrödinger equation for a one-dimensional, coupled two-level system is^1,63,64^
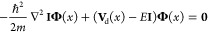
1where **I** is the 2 × 2 identity matrix; **V**_d_ is a 2 × 2 real symmetric, nondiagonal, diabatic potential energy matrix with two potential energy curves (PECs) *V*_1_(*x*) and *V*_2_(*x*) as diagonal elements, and an interaction potential *V*_12_(*x*) on both the off-diagonals; **Φ**(*x*) is the two-component vector of solutions ϕ_1_(*x*) and ϕ_2_(*x*); *m* is the mass of the system and *x* the coordinate. We use atomic units such that ℏ = 1.

Following Baer,^1^ we define the adiabatic state vector **Ψ**(*x*) as a vector of the two states ψ_α_(*x*) and ψ_β_(*x*), such that it is related to **Φ**(*x*) by a transformation matrix **A**(*x*) (suppressing the *x*-dependence notation from here):
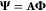
2**A** is an *x*-dependent matrix that diagonalizes the diabatic matrix **V**_d_(*x*) to produce the diagonal adiabatic matrix **V**_a_(*x*) with elements *V*_α_(*x*) and *V*_β_(*x*) (**V**_a_ = **AV**_d_**A**^†^). In this notation, the adiabatic representation of the TISE is
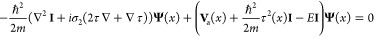
3where σ_2_ (also σ_*y*_) is the second Pauli matrix and τ(*x*) is a function that determines the strength of the nonadiabatic coupling between the two adiabatic states. This equation is equivalent to eq 1. τ(*x*) serves two roles in this equation: it defines the off-diagonal coupling between the two adiabatic states, and it adds an extra diagonal contribution, which we will refer to as the nonadiabatic coupling term, NACT:
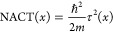
4(Note the slightly nonstandard notation: NACT often refers in the literature to τ itself.)

The adiabatic PECs *V*_α_(*x*) and *V*_β_(*x*) can be expressed in terms of the diabatic PECs:
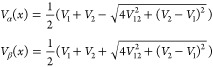
5

We investigate three cases: the numerically exact dynamics of eq 1 (which we compute in the diabatic picture) and two levels of approximation: the fully adiabatic dynamics of eq 3 defined by setting τ(*x*) = 0 and an intermediate case where the *off-diagonal* contributions of τ(*x*) (in the first parentheses of eq 3) are ignored, but the on-diagonal contribution is kept: an “adiabatic+NACT” approximation (or simply “NACT” for short). The latter two approximations have no couplings between the adiabatic states and are thus simple one-level systems with two possible exit channels for the scattering event: transmission and reflection. The numerically exact case refers to the solution of the coupled dynamics in the diabatic picture with no approximations, so that the two-level system has in principle four possible exit channels (some of which will be closed at sufficiently low scattering energy).

τ(*x*) itself is defined in terms of the rotation angle β(*x*) that rotates the state **Φ** into the state **Ψ**. It is the negative of the derivative of β(*x*), where
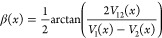
6Hence it can be shown that^1^
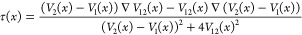
7

In the single avoided crossing model of Tully,^51^ the diabatic terms in the potential matrix are (see Figure 1)
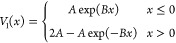
8
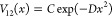
9and *V*_2_(*x*) is obtained by the transformation *x* → (−*x*) everywhere in eq 8. Here, *A* = 0.01, *B* = 1.6, *C* = 0.005, *D* = 1.0, ℏ = 1, and *m* = 2000 atomic units (all quantities will be expressed as such from now on).

**Figure 1:**
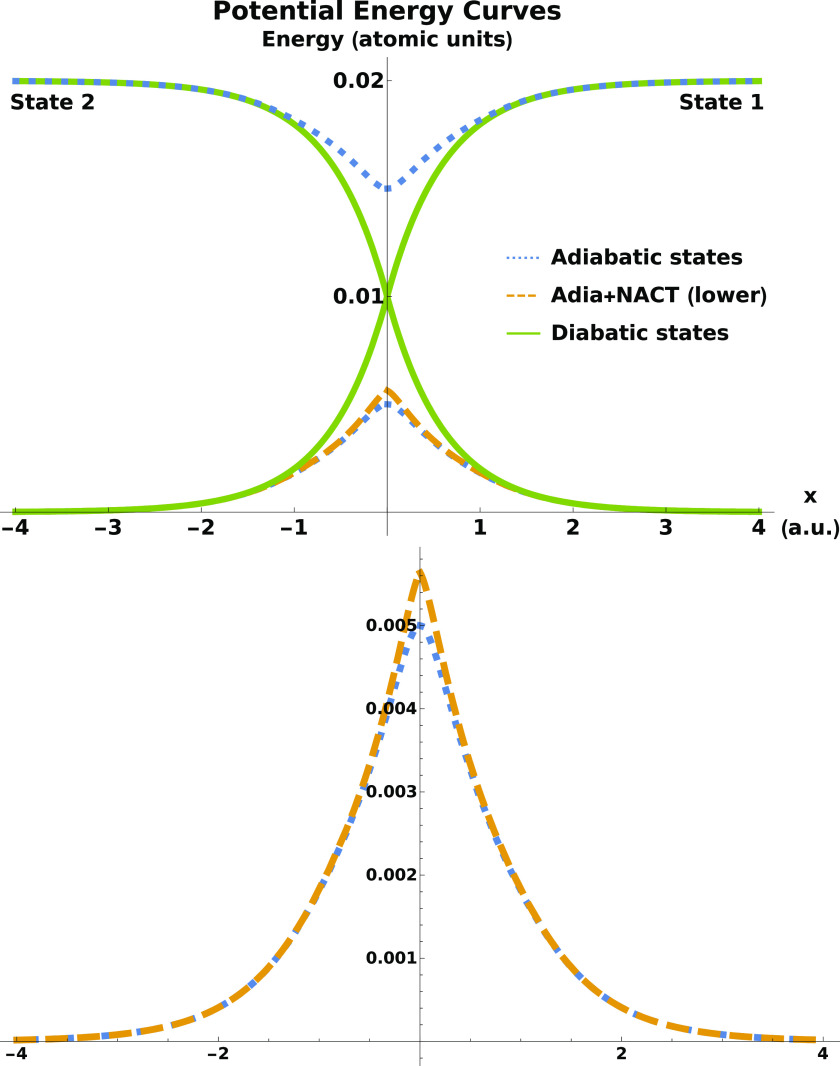
Potential energy curves, from Tully’s single avoided crossing model. Upper: the two diabatic curves and two adiabatic curves are presented, along with the lower adiabatic+NACT curve (the energy regime in which the upper adiabatic curve is accessible is not studied here and is presented only for reference). The lower adiabatic curve peaks at 0.005 atomic units, but with a nonadiabatic correction added it peaks at 0.00564 atomic units. (The off-diagonal diabatic coupling *V*_12_(*x*), if plotted, would peak at the same point as the adiabatic curve, but it would be slightly wider near the top.) Lower: close-up of the lower adiabatic curve and lower adiabatic+NACT curve.

To study the tunneling time, we focus exclusively on energies below the threshold for the opening of the upper state such that the scattering energy *E* is always less than 0.02. Therefore, no transmission or reflection is possible on the upper state (which is diabatic state 1 on the right and diabatic state 2 on the left); they are closed channels. We compute four different time quantities: the transmitted and reflected phase times τ_φ,(*T*,*R*)_(*E*) and the transmitted and reflected tunneling flight times *t*_TFT,(*T*,*R*)_(*E*) (as outlined in ref (45) based on refs (40−42 and 44)).

In all results shown here, the initial, incoming amplitude of the wave function was to the left of the interaction region, on the lower state (*V*_1_(*x*) in the diabatic case, *V*_α_(*x*) in the adiabatic case, and *V*_α_(*x*) + NACT(*x*) in the NACT case). Times are computed using both time-dependent and time-independent methods. The time-dependent method involves propagating a wave packet with initial position *x*_i_, initial momentum ℏ*k*_i_, and initial spatial width *s*_i_ (the functional form being ). The wave packet is propagated through the interaction region, and the mean transition time is extracted from the final time distributions on both the right and left of the interaction region. The process is repeated for a number of wave packets of different widths, and the limits are taken as the spatial widths tend to ∞ (and thus the momentum widths to zero). This limiting procedure gives the transmitted tunneling flight time *t*_TFT,*T*_(*E*) and its reflected counterpart *t*_TFT,*R*_(*E*) (see eq 9 in ref (45)).

When using the time-independent method, we calculate the phase times, quantities that are defined only for plane-wave scattering at a single energy, not a distribution of energies. The scattering wave function has an incoming component on the lower surface to the left and has four possible scattered components, each with associated scattering amplitudes: transmission and reflection on the upper and lower surfaces. Since the upper surfaces are energetically closed, there are in practice two scattering amplitudes to consider: lower transmission *T*(*E*) and lower reflection *R*(*E*). The transmission and reflection probabilities are then given by |*T*|^2^ and |*R*|^2^, respectively. The transmission amplitude (or equivalently reflection amplitude) is expressed as *T*(*E*) = |*T*(*E*)|exp(*iφ*_*T*_(*E*)), and this defines the transmission phase. The energy derivative of the phase, φ_*T*_(*E*), gives the transmitted phase time (and similarly the reflected phase time):
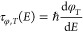
10

For the time-dependent method, the tunneling flight time^45^ was calculated as the difference between the mean time-of-flight it takes the incident wave packet to reach a screen located outside of the interaction region, minus the time it would take a free particle with the same incident wave packet to reach the screen, with corrections for momentum-filtering,^22,25,65^ as described in refs (44 and 45).
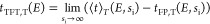
11We have previously demonstrated^44^ that the tunneling flight time *t*_TFT,T_(*E*) is identical to the phase time.

Computationally, the time-independent numerically exact results are obtained using the log-derivative method,^66^ with the boundary conditions as given in ref (67) and using the propagator as described in refs (68 and 69). The adiabatic results follow the same numerical scheme as in previous works^44,45^ based on ref (70) and using Mathematica’s numerical differential equation solving methods (the asymptotic regions were all set to be at ±15 atomic units). These algorithms provided the transmission and reflection amplitudes. Numerical derivatives were obtained using the five-point stencil method of numerical differentiation with an energy spacing of 5 × 10^–5^ atomic units (2 × 10^–6^ in the resonance region). For each of the time-independent results shown, the error bars would be too small to visualize on the plots.

The time-dependent results were obtained using the split-operator method for wave packet propagation, implemented in a modified version of the code *wavepacket*(71) that allows for two-level systems and potentials. For each of the plotted flight time points, mean flight times were calculated for four different wave packet widths (*s*_i_ = { 14.1, 15.8, 18.3, 22.4}) and then extrapolated linearly to zero width. This zero-width limit of the time is the quantity plotted in the figures below. Each wave packet was initially centered around *x*_i_ = −100 and propagated toward a screen at *Y* = 200, with a further screen at *Y* + *ΔY* = 224. The eight mean initial momenta were *k*_i_ = {3.16228, 3.4641, 3.74166, 4.0, 4.24264, 5.38516, 6.2450, 7.0} (a.u.). The spatial grid spacings used for the split-operator propagation were 0.005, the time steps were 0.8, the *x*-range was taken to be from −690 to 690, and the *t*-range was taken to be from 0 to 1.5× the free classical time-of-flight for a given momentum ℏ*k*_i_.

The split-operator method is prone to finite boundary effects, but error testing revealed that the grid spacings were sufficiently small and the *x*-range sufficiently large to account for this (likewise, the time steps were sufficiently small and the initial position sufficiently far from the interaction region). For both the transmission probabilities and the flight times, the time integration used to obtain the plotted results was performed over the density distributions rather than the flux distributions. Results from flux distributions were tested and shown to be negligibly different from results based on the density distributions, as also observed in our previous work.^44^ The integrations themselves were found to produce errors in the range of 4 × 10^–6^, which corresponds to a time uncertainty of approximately one atomic unit in the calculations of the flight times, though this is likely to lead to errors approximately two or three times larger than that when taking differences between flight times.

There are three distinct energy regions one can demarcate below *E* = 0.02. The lowest-energy region is the “tunneling” region (0 < *E* < 0.005). For the adiabatic and NACT approximations, the dynamics in this regime is effectively that of a one-dimensional tunneling problem whose dynamics is similar to the systems studied in several previous papers.^43−46^ The second region (0.005 < *E* < 0.015) may be referred to as the “above-barrier” region: within the adiabatic approximation this energy region lies between the top of the barrier of the lower adiabatic state and the bottom of the upper adiabatic state. Here the physics is dominated mostly by transmission. The third region (0.015 < *E* < 0.02) is the “resonance” region. In the adiabatic and NACT cases, there is a potential energy well in the upper state, which supports several bound states. This manifests itself in the numerically exact solution of the dynamics as a series of Feshbach resonances^72,73^ in the transmission probability, which are also present in plots of the phase time.

Figure 2 plots the energy-dependent transmission probabilities |*T*|^2^ as functions of the energy spanning all three energy regions. Results are shown for the adiabatic, NACT, and numerically exact transmission probabilities. The probabilities are calculated using two methods: time-dependent wave packet propagation (circles) and time-independent log-derivative methods (curves), with the proviso that the time-dependent calculations are not performed in the resonance region. In the tunneling and above-barrier regions, nonadiabatic couplings are seen to suppress transmission probabilities. This is a consequence of the barrier height being slightly increased by the NACTs, as seen in Figure 1. Deviations between the two sets of results never exceeded a relative error of 4 × 10^–5^; the time-dependent wave packet results were the same as the time-independent plane-wave results when the appropriate zero-width limit was taken. This agreement serves also as a check of the accuracy of the numerical results presented. It is important, especially due to the fact that some differences induced by the nonadiabatic coupling were relatively small.

**Figure 2:**
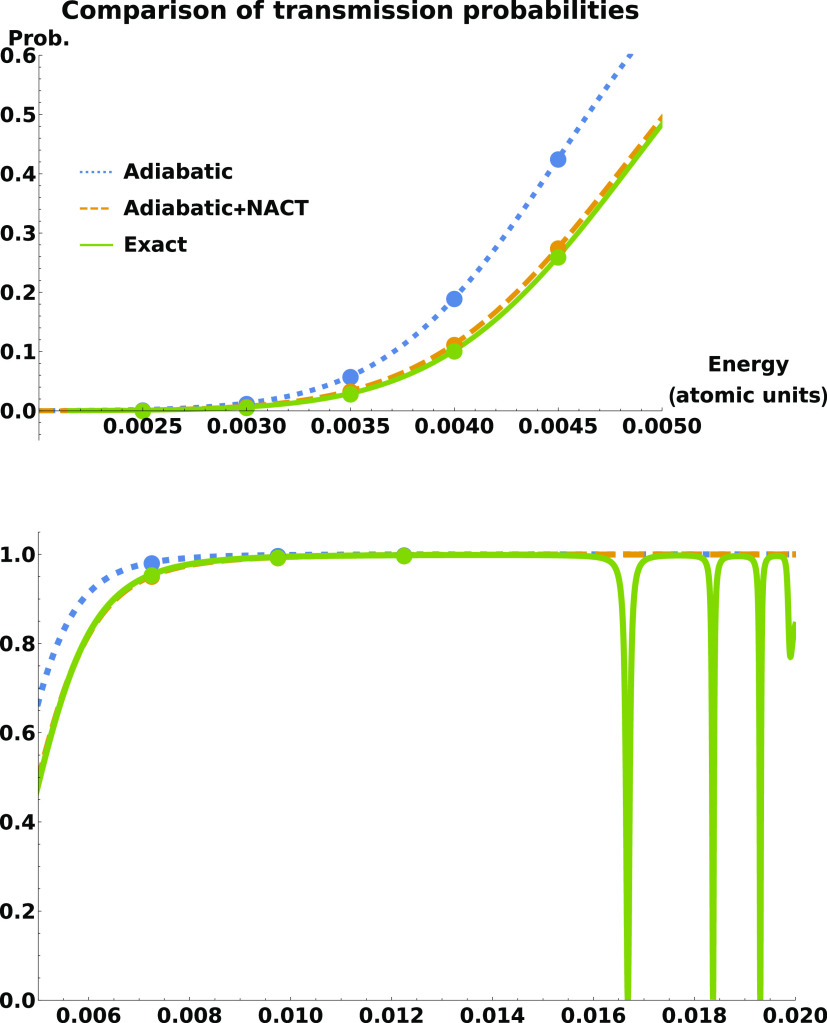
Transmission probabilities for three different computations (adiabatic, adiabatic+NACT, and numerically exact) for the three energy regions: tunneling (top), above-barrier, and resonance (bottom). Results from time-independent calculations with plane waves are given by the curves, and results from time-dependent wave packet propagation are given by circles. The relative error between the two calculation methods never differs by more than 4 × 10^–5^. The resonance positions match those in our previous work;^48^ however, the resonance widths are much smaller due to the lack of energy averaging of an initial wave packet.

The positions of the Feshbach resonances are the same as those presented in the Supporting Information of ref (48), where the values were calculated using a different method. This serves as an additional independent check on the validity of the results presented here. The main difference between the results plotted in Figure 2 and the previous results is that here we show the energy-dependent results rather than those obtained from wave packets with a finite momentum width, which leads to a “smearing” of the resonances. We find that on resonance, there is a pure reflection of the incident plane wave. This is in contrast to the pure on-resonance transmission found for example for a square well potential bounded by two barriers.^74^ In this avoided crossing case, the analogous transmission channel on the upper surface is closed, and therefore, on resonance one finds pure reflection.

The central result of this Letter, which is that the nonadiabatic coupling reduces the tunneling flight time, is shown in Figure 3 and in further detail in Figure 4. Figure 3 shows the reflected and transmitted times of flight on the lower states as functions of scattering energy, calculated both time-independently as the phase time (curves) and time-dependently as the tunneling flight time (circles and diamonds). There is no need to plot the reflected phase times as they are identical to their transmitted counterparts—for any smooth symmetric potential the two coincide.^45^ The mean times plotted in the figure are shown for all three energy regimes. The numerical deviation between time-dependent and time-independent results is such that no tunneling flight time deviated from its respective phase time by more than 6 atomic units of time. The somewhat larger deviation of times as compared to probabilities should be understood in the context that the tunneling flight time is a difference between two much longer times each of the order of 2 × 10^6^.

**Figure 3:**
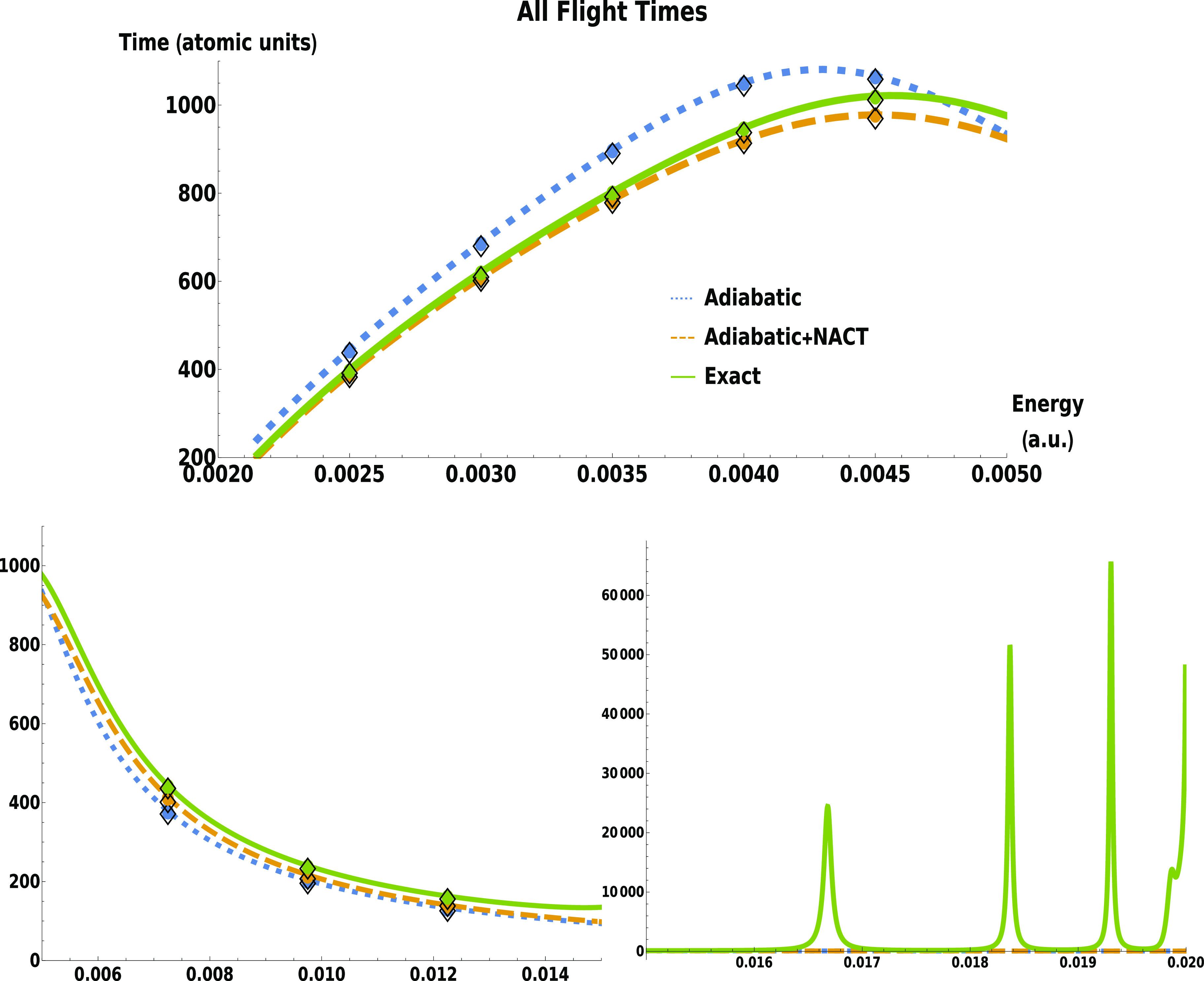
Transition path flight times plotted as a function of energy in the three energy regions: tunneling (top panel), above-barrier (bottom left panel), and resonance (bottom right panel). The transmission results from time-independent calculations on plane waves (phase times, τ_Φ,*T*_) are given by curves. Transmission results from time-dependent calculations on wave packets (tunneling flight times, *t*_TFT_) are given by circles, and time-dependent reflection results are given by diamonds. Each circle and diamond deviates from its respective line by no more than 6 atomic units of time. The unplotted reflected phase times deviate from the transmitted phase times by no more than 0.008 atomic units (except near resonances, where numerical differentiation cannot be relied upon to the same extent).

**Figure 4:**
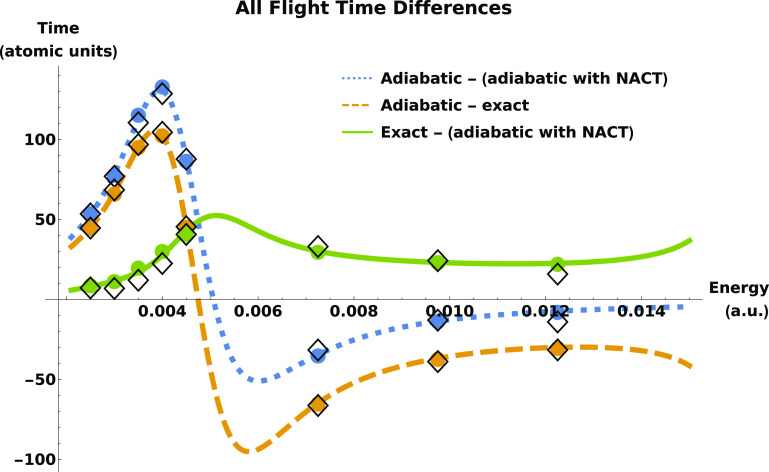
Differences between flight times are plotted as functions of the energy in the two lower-energy regions. Transmission results from time-independent calculations on plane waves (phase times) are given by curves, and transmission (circles) and reflection (diamonds) results from time-dependent wave packet propagation (tunneling flight times) are given by points.

It is interesting to note that in the resonance region, there appears to be a hint of a fifth resonance right below the opening of the upper threshold at 0.02 atomic units of energy, something which is not seen in the transmission probability. This could be due to a fifth, rather shallow bound state in the upper potential well (this possibility was not ruled out in our previous work^48^). There is though the possibility that it is a numerical artifact of the methods used—the asymptotic region may not have been placed far enough out to treat the channel as properly closed just below the threshold, leading to a “tunneling-like” effect where there is none physically.

Also, we note that the times at the peaks of the resonances—of the order of 10^4^ atomic units—are similar to those calculated in the “narrow wave packet” case in our previous work. In contrast, in terms of transmission probabilities, our previous work did not detect resonances strong enough to fully reflect the incoming wave.

To accentuate the effect of the nonadiabatic coupling on the tunneling flight times we plot in Figure 4 differences in the flight times: adiabatic minus numerically exact, adiabatic minus adiabatic with NACTs, and numerically exact minus adiabatic with NACTs. The resonance energy region is not shown because it is outside the tunneling energy regime which ends at 0.005 atomic units in the adiabatic case and 0.00564 when the NACTs are added. The small differences between the time-dependent and energy-dependent generated results are due to numerical error only.

The key result, and the headline of this work, is that tunneling is slower when the system is adiabatic, compared to when some or all of the diabatic couplings are included. The dynamics is reflection-dominated (|*T*|^2^ < 0.5) until approximately *E* = 0.005 atomic units. In this energy region, the tunneling times as obtained from the adiabatic computations are larger than those obtained from the numerically exact computation. Above *E* ≈ 0.005 au, |*T*|^2^ > 0.5 and the dynamics is dominated by transmission. In this energy regime, adiabatic times are lower than nonadiabatic ones.

The comparisons considered in Figure 4 of the three time computations show that most of the deviation from the adiabatic times comes from adding the on-diagonal NACT to the adiabatic potential energy surface. The off-diagonal nonadiabatic couplings present in the numerically exact computation do not contribute to the altering of the tunneling times as strongly. Notably, the line “Exact – (adiabatic with NACT)” is always positive: adding the off-diagonal nonadiabatic couplings slows down the flight time slightly in all cases. The major contribution to the shortening of the tunneling time comes from the diagonal term: it increases the height of the adiabatic barrier. This can be understood as the density under the barrier being reduced, which serves to shorten the tunneling time. Similar results were found in ref (37) in the context of Larmor times, which are strongly connected to dwell times, which depend on the density under the barrier. It is also noteworthy that, as seen from the top panel of Figure 2, the nonadiabatic coupling reduces the transmission probability in the low-energy region as compared to the two adiabatic computations.

One may question whether the results presented here have any practical implications. It is too early to give a definitive answer; however, we do note that recent experiments on tunneling times^36,37^ have demonstrated the ability to measure (Larmor) phase times. A central result of this Letter is the observation that the phase time is the correct measure to use also when considering nonadiabatic transitions, pointing out that here too it is equivalent to the flight time in the appropriate limit. At least in principle, the formalism used here demonstrates that time-of-flight experiments may reveal interesting aspects of nonadiabatic effects. Second, the time analysis provides a benchmark with which to test approximate theories and understand their advantages and shortcomings. Third, the effect presented here, which is that nonadiabatic coupling affects tunneling times, might be even more dramatic when treating multidimensional systems, especially in the context of conical intersections. In this context, we note that closed channels must often be included when evaluating tunneling transmission probabilities; do they also affect the tunneling times? If coupling to another energy level can result in speed-ups of tunneling events, would this have implications for the MacColl–Hartman effect and other peculiarities of quantum tunneling times?

It would also be of interest to understand whether spin can have an effect on tunneling times. Will they be faster or slower for bosons or fermions? The present study is perhaps a first step in consideration of tunneling times in multidimensional systems.
